# Molecular docking analysis of triterpenoids from *Cassia fistula* with breast cancer targets

**DOI:** 10.6026/973206300191067

**Published:** 2023-11-30

**Authors:** Ireen Christopher, Aishwariya Sounderraajan, Viji Murugesan, Indu Sabapathy, Vijayalakshmi Periyasamy, Rajalakshmi Manikkam

**Affiliations:** 1 Department of Biotechnology and Bioinformatics, Holy Cross College (Autonomous), Tiruchirappalli, Tamil Nadu, India; 2 DBT-BIF Centre, Holy Cross College (Autonomous), Tiruchirappalli, Tamil Nadu, India; 3 Department of Zoology, Holy Cross College (Autonomous), Tiruchirappalli, Tamil Nadu

**Keywords:** Breast cancer, anticancer, chemo-resistance, *Cassia fistula*, triterpenoids

## Abstract

Breast cancer is a well-known complex disease. The availability of different screening approaches and booming phytochemical drug synthesis can contribute towards breast cancer treatment. Hence, we document the molecular docking analysis of triterpenoids from *Cassia fistula* with breast cancer targets.

## Background:

There are many naturally derived chemotherapeutic drugs available for cancer [1]. *Cassia fistula* (*C. fistula*) belonging to the Caesalpiniaceae family is one such natural source of traditional medicines followed in Unani, Ayurveda, and Chinese treatment. *C. fistula* native to South Asian continents are cultivated in various parts of the world for their constituents such as triterpenes, sugar, rhein and potassium [2]. Studies based on the methanolic extracts of the *C. fistula* seeds showed a decrease in viable tumour cell count [3]. The anticancer efficacy of different fruit extracts of *C. fistula* against human cervical cancer (SiHa) and breast cancer (MCF-7) cell lines proved the upregulation of apoptotic markers [4]. Among the physically diverse family of secondary metabolites known as isoprenoids, terpenoids are the largest subfamily and are categorised according to their pentyl count. More than 14,000 structures have so far been documented [5]. According to Alqahtani, A. et al. (2013) [6], most triterpenoid compounds have 30 carbon atoms and are made up of 6 isoprene units of mevalonic acid or deoxy-xylulose phosphate. The triterpenoids can be categorised as follows based on their fundamental carbon skeleton [7]. Pentacyclic triterpenoids are the main family of chemical compounds derived from naturally occurring plant materials and can be found as free acids, esters, or glycosides (saponins) [8]. According to Xu C et al. (2017) [7], several therapeutic herbs used in Chinese medicine include pentacyclic triterpenoid chemicals. According to [9, 10, 11, 12, 13] triterpenoids are commonly found in marine sponges, vegetables, fruits, grains, and spices. Triterpenoids are used in a variety of applications, including surface waxes, specialised membrane chemicals, and signalling molecules [14]. To protect the body has been infections and xenobiotics, triterpenoids are produced [15]. Triterpenes isolated from various plants are reported to have various pharmacological applications which includes immunomodulatory, Anti-tumour, anti-proliferative, anti-oxidant and anti-inflammatory. Breast cancer is affecting women all around the world, with highest mortality and incidence rates. Since the last Consensus conference in 2019, breast cancer has surpassed lung cancer and is now the cancer type with most incidence and mortality rate [16]. In 2020, it is the most commonly diagnosed cancer with an estimate of around 2.26 million cases around the world, and is the leading cause of mortality due to cancer in females [17]. Breast cancer is the common cancer that affects women around the world [18]. It is also the type of cancer which has different presentations among [19]. The treatments for breast cancer involve, surgical removal of breasts, radiation therapy, chemotherapy, and so on. The need of the hour is for a treatment plan with less side effects with higher quality of life for the women with the incidence or at the risk of incidence of breast cancer. One of the common and widely used treatment methods for breast cancer is adjuvant chemotherapy, but even with that the five-year survival rate is less than 30% [20]. Paclitaxel being one of the staple treatment drugs for cancer treatments has been reported to be risking the quality of life for the women with side effects. About 6, 85,000 deaths were reported in the year 2020 amongst females worldwide [17]. Different kinds of biomarkers for diagnosis, prognosis, drug resistance, and therapeutic implications have been found thanks to molecular technologies. The commonly used biomarkers are the apoptotic proteins, cell cycle proteins, NFkB proteins, WTN proteins and oxidative stress markers; these are responsible for the upregulation and downregulation of the tumour. Some are also responsible for the malignancy, stemness and drug resistant properties of cancer. The use of these biomarkers could help in addressing the issue of drug-resistance in the treatment of breast cancer [20]. Therefore, it is of interest to report the molecular docking analysis of triterpenoids isolated from *Cassia fistula* with cancer targets.

## Materials and methods:

## Receptor preparation:

The 3D X-ray crystallographic structures of the target proteins were obtained from Protein Data Bank (PDB) (Table 1). The receptors were prepared by removing the hetero-atoms and water molecules and adding polar hydrogen atoms using the Discovery Studio Visualizer 2017 R2 Client software.

## Ligand preparation:

Using ACD labs' Chemsketch, 3D structures for Triterpenoid Compound1, Triterpenoid Compound 2, Triterpenoid Compound 3, were created. The ligands that were drawn and imported in MOL format were translated in the PyRx tool into PDBQT files. The target protein's binding site could be prepared and a chemical library could be screened using PyRx Version 0.8, which was used to dock the receptor proteins and their ligands [21]. Software called Discovery Studio 2017 R2 Client was used to display the results [22].

## Drugability:

Drugability properties like Lipinski rule of five (Table 2) and ADMET profiling (Table 3) of the ligands were analysed using pkCSM online pharmacokinetic tool [23].

## Molecular docking:

**Figure 1:**
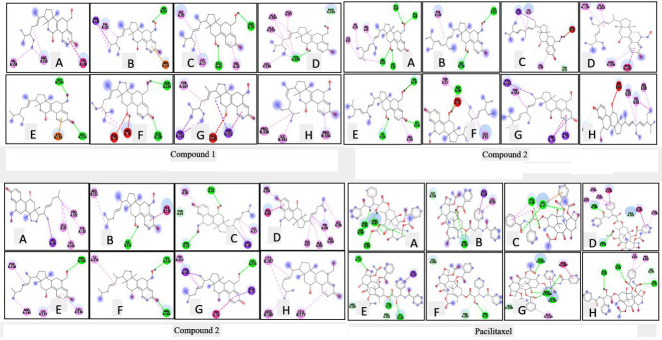
Molecular interaction of tetracyclic triterpenoids (a,b,c) and paclitaxel (d) with apoptotic markers (A)BAK (B)BAX (C)BCL-2 (D)BCL-xL (E) Caspase-3 (F) Caspase-6 (G) caspase-8 (H) caspase-9

**Figure 2:**
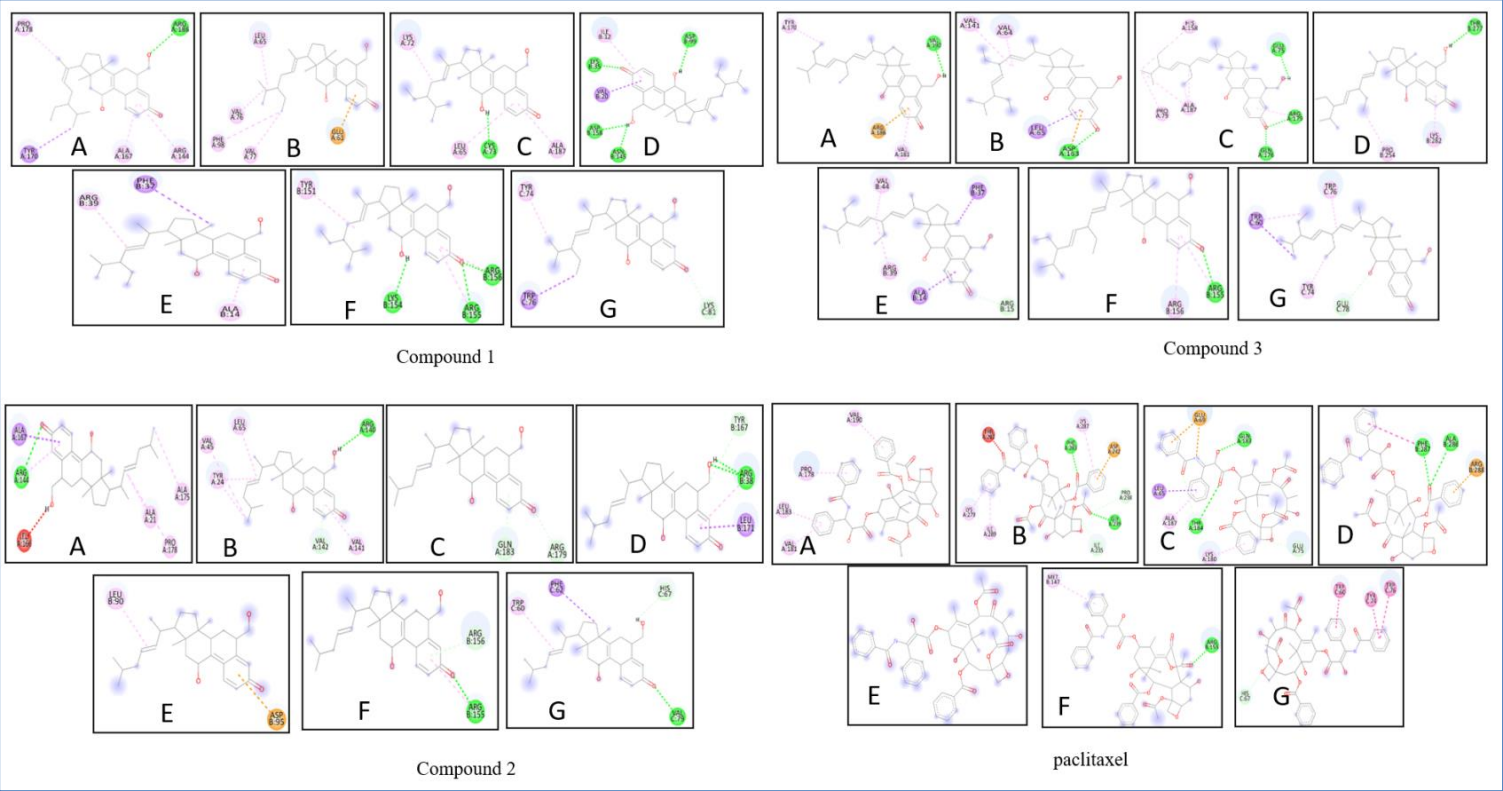
Molecular interaction of tetracyclic triterpenoids (a,b,c) and paclitaxel (d) with cyclic proteins (A) CDK4 (B) CDK6 (C) Cyclin D1 (D) Cyclin D3 (E) P18 (F)P21 (G) p^27^

**Figure 3:**
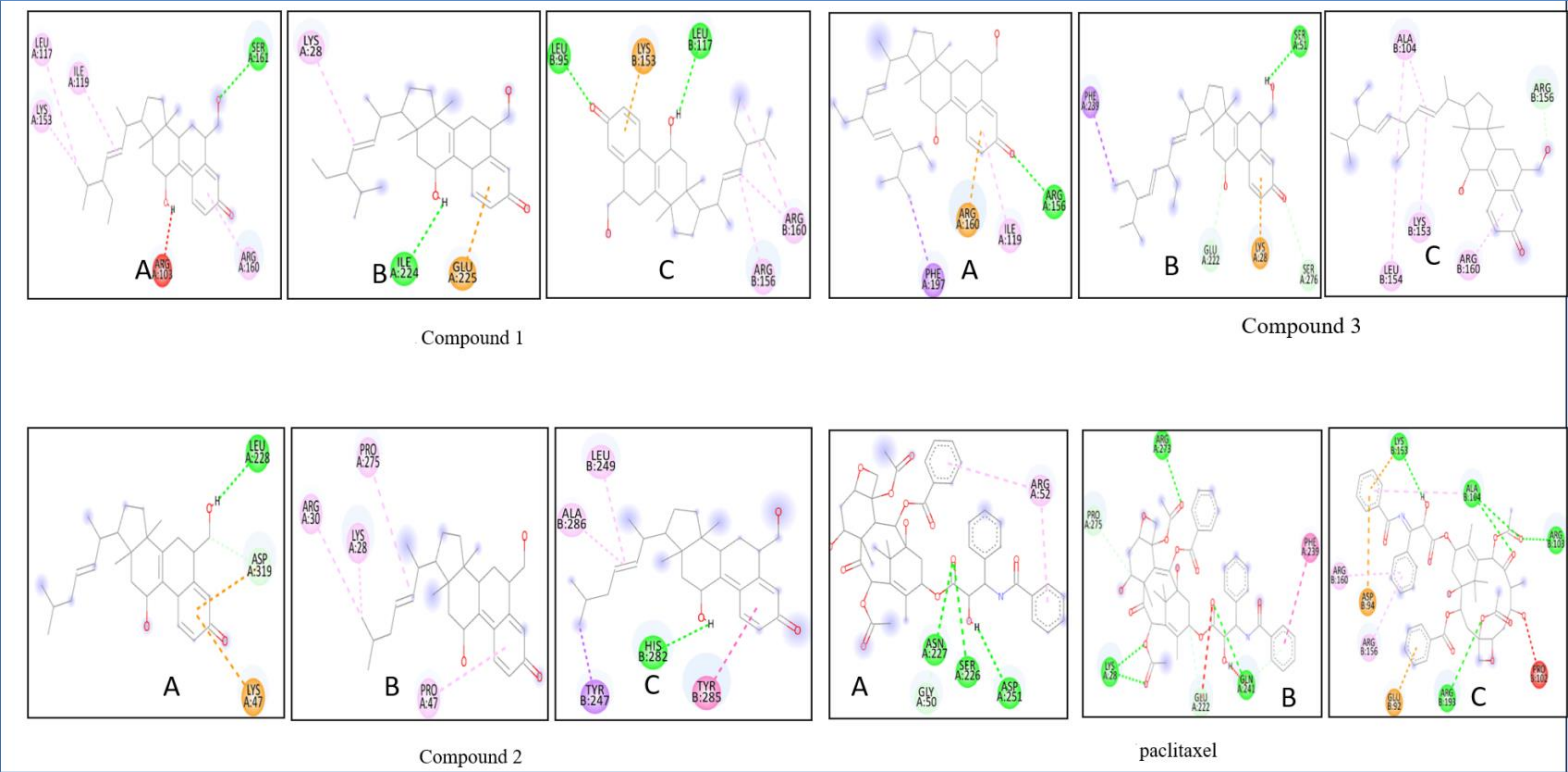
Molecular interaction of tetracyclic triterpenoids (a,b,c) and paclitaxel (d) with NFkB proteins (A)NFkB-p^52^ (B)NFkB-p^65^ (C) NFkB-p^100^

**Figure 4:**
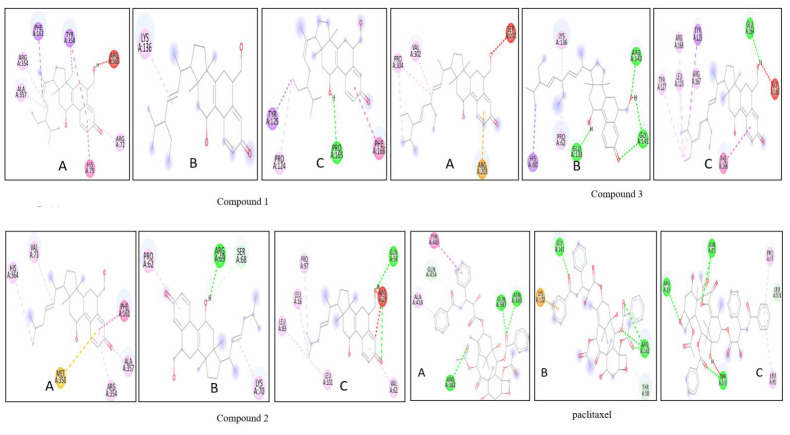
Molecular interaction of tetracyclic triterpenoids (a,b,c) and paclitaxel (d) with Oxidative stress markers (A)CAT (B)SOD (C) GPx

**Figure 5:**
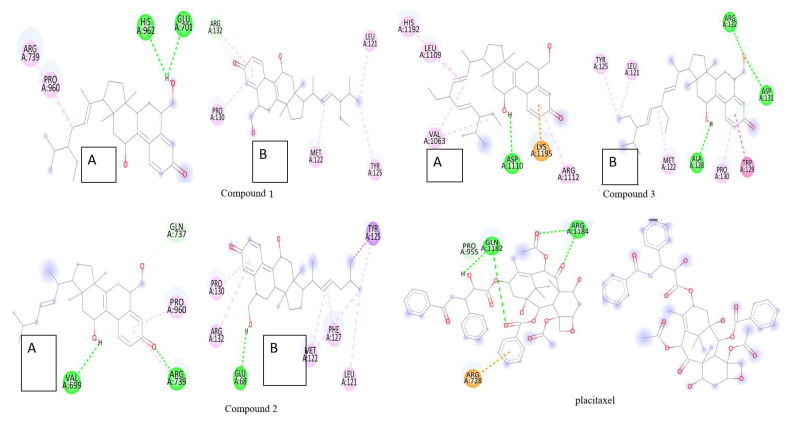
Molecular interaction of tetracyclic triterpenoids (a,b,c) and paclitaxel (d) with WNT proteins (A) LRP (B) WNT Frizzeled

The docking studies were achieved by PyRx (Auto dock vina) tools version v0.8 programs. To the ligand moieties polar hydrogen was added and the searching grid extended above the preferred target proteins. Atomic solvation parameters and Kollman charges were added. Non-polar hydrogen atoms were merged with the carbons and the internal values of torsions were adjusted along with the polar hydrogen charges of the Gasteiger-type. The search was carried out with the Lamarckian Genetic Algorithm. Affinity maps for all the atom types present, as well as an electrostatic map, were computed with a grid spacing of 0.375 Å. Evaluation of the results (Figure 1,Figure 2,Figure 3 Figure 4,Figure 5) was done by sorting the different complexes with respect to the predicted binding energy. A cluster analysis based on root mean square deviation values, with reference to the starting geometry, was subsequently performed and the lowest energy conformation of the more populated cluster was considered as the most trustable solution. The hydrogen bond atoms involved in the molecular docking along with the binding affinity for each is noted down below in Table 4.

## Results and Discussion:

Methods for determining molecular properties and bio-pharmaceutic predictions for developing new medication candidates are available. Lipinski's rule of five, a widely used way to forecast a drug's ADME ("absorption, distribution, metabolism, and excretion") performance, is a broad "rule of thumb" for valuing drug-like features that has been around for about 20 years [24]. The triterpenoids isolated from *C. fistula* when subjected to drug-ability profiling showed promising scaffold for Lipinski rule of five. When compared to the reference drug paclitaxel, all the three triterpenoids follow the Lipinski rule of 5 (Table 2). Similarly, ADMET profiling of the triterpenes and comparing them to paclitaxel showed the drug-ability of the triterpenes (Table 3).

Subjecting the triterpenoids and the reference drug paclitaxel to molecular interaction with different cancer targets like apoptotic (Caspase-3, Caspase-6, Caspase-8, Caspase-9), pro-apoptotic (BAK, BAX, Bcl-2, Bcl-xL), Cell cycle (CDK-4, CDK-6, Cyclin D1, Cyclin D3, p^18^, p^21^, p^27^), NFkB (p^52^, p^65^, p^100^), WNT (FZD, LRP) and oxidative stress (CAT, SOD, GPx) showed good covalent interaction with good binding affinity (≥ -5) among which Triterpenoid Compound 2 has better binding affinities with more hydrogen bond interactions. The entrance to the mitochondrial route of apoptosis is through Bax and Bak (Table 4).

According to research employing truncated Bak molecules, the truncated molecule still can bind to Bcl-xL but lacks the membrane anchoring region. Increases in BAK protein levels brought on by gene transfer speed up the apoptosis that growth factor deprivation causes in breast cancer cells [25]. There are two main pathways that trigger apoptosis: the extrinsic [or death receptor (DR)] pathway is triggered by ligand binding of DRs superfamily members, which causes caspase-8 and caspase-3 to be activated; the intrinsic (or mitochondrial) pathway is triggered by mitochondrial release of cytochrome c, which causes Apaf-1 and cytochrome c complex to form with the help of ATP, which then activates caspase-9 and capase-3 [26]. The initiator caspases (caspase-2, -8, -9, and -10), and the effector caspases (caspase-3, -6, and -7), are two subgroups of the typical apoptotic caspases [27]. Thus, Figure 1 depicts the molecular interaction occurring between the ligands (triterpenoid compounds and paclitaxel) and the apoptotic markers, in which the binding affinity for the triterpenoid compounds (a,b,c) is significantly less than the binding affinity for paclitaxel (d), whereas the hydrogen bond interaction is significantly more prominent in triterpenoids rather than paclitaxel.

A variety of growth cues during G1 can cause cyclin D to bind to CDK4 or CDK6, which leads to the phosphorylation of Rb and eventual release of E2F and cell cycle advancement [28]. One of the three cyclin D proteins, cyclin D3 is overexpressed in a variety of human malignancies, including breast cancer. The oncogenic significance of cyclin D3 in cancer was demonstrated by the inhibition of cyclin D3 expression in mammary tumor cells, which inhibited cancer cell proliferation in vitro and decreased the tumor burden in vivo. It has been demonstrated that the phosphorylation of cyclin D3 by proteasomes regulates the levels of cyclin D3 inside the cell [29]. Cell cycle regulator p21 was first identified as a CDK inhibitor with the capacity to cause growth arrest by inhibition of Cdks, which are necessary for the G1 to S transition. Cell cycle regulator p21 is a protein encoded by the CDKN1A gene, which is also known as the CDKN1A gene. Because cell cycle regulation is closely related to carcinogenesis, the role of p21 in the development of carcinomas has attracted a lot of attention. High p21 expression has been linked to poor prognosis in clinical research, and some studies have suggested that CDKN1A/p21 promotes cancer growth and may possibly be a factor in drug resistance [30]. Figure 2 represents the molecular interaction between the triterpenoids and paclitaxel with the cyclic proteins, in which the binding affinity and hydrogen bond formation are desirable towards the triterpenoids (a,b,c) when compared to paclitaxel (d). NF-B signalling is crucial for the development, spread, and metastasis of cancer. By targeting the cyclin D1 gene, RANKL activates NF-B in breast cancer, causing cellular proliferation. Increased levels of Bcl-xL and inhibitors of apoptosis (IAPs) are another way that NF-B mediates survival [31]. Figure 3 illustrates the molecular interaction with binding affinity and hydrogen bon interaction with the triterpenoids (a.b.c) and paclitaxel (d) with NFkB proteins, in which the binding affinity and hydrogen bond formation is favourable for the triterpenoids. ROS can cause programmed cell death due to their damaging effects on DNA, proteins, and the integrity of the plasma membrane [32]. Figure 4 outlines the molecular interaction of the ROS proteins with the triterpenoids (a,b,c) and paclitaxel (d), in which the triterpenoids have agreeable outcomes. One of the most evolutionary conserved routes, Wnt signalling is crucial for many biological functions, including embryonic development and adult tissue homeostasis. The pathophysiology of numerous human malignancies is characterised by dysregulation of the Wnt pathway [33]. The molecular interaction of the WNT signalling proteins with the triterpenoids (a,b,c) and paclitaxel (d) which outlines the favourable conditions the triterpenoids provide in the interaction rather than paclitaxel. Thus, the triterpenoids can be considered for further implications regarding breast cancer treatments.

## Conclusion:

The molecular interaction between the markers and the triterpenoid compounds is documented. There was more hydrogen bond interaction with the reference drug paclitaxel. The triterpenoids showed better binding affinities and better scaffold for binding. The triterpenoids can be a better alternate for the treatment plans of breast cancer patients and can be subjected to further studies in order to facilitate more knowledge about the exact action of these triterpenoids against the breast cancer targets.

## Figures and Tables

**Table 1:** Apoptotic, Cell Cycle, NFkB, oxidative stress markers and WNT, proteins along with their PDB IDs and chains considered in this study

**Receptors**	**PDB ID**	**Chain**
Cyclin Dependant Kinase- 4 (CDK-4)	3G33	A
Cyclin Dependant Kinase- 6 (CDK-6)	1G3N	A
Cyclin-D1	2W99	A
Cyclin-D3	3G33	B
Cyclin Dependant kinase inhibitor 4c (p18Ink4c)	1G3N	B
Cyclin Dependant kinase inhibitor 1 (p21WAF1/Cip1)	1AXC	B
Cyclin-dependent kinase inhibitor 1B (p27Kip1)	1JSU	C
B-cell leukemia/lymphoma 2 protein (BCL-2)	1G5M	A
B-cell lymphoma-extra-large (Bcl-xL)	1G5J	A
BCL-2 antagonist/killer (BAK)	2YV6	A
BCL-2-associated X protein (BAX)	2K7W	B
Caspase-3	1GFW	A
Caspase-6	2WDP	A
Caspase-8	5JQE	A
Caspase-9	1NW9	B
Nuclear factor NF-kappa-B p52 subunit	1A3Q	A
Nuclear factor NF-kappa-B p65 subunit	1NFI	A
Nuclear factor NF-kappa-B p100 subunit	3DO7	B
Catalase (CAT)	1QQW	A
Super-oxide Dismutase (SOD)	1SPD	A
Glutathione Peroxidase- 2 (GPx-2)	2HE3	A
Low Density Lipoprotein Receptor-Related Protein (LRP)	4A0P	A
Frizzled Protein (FZD)	6AHY	A

**Table 2:** Comparison of the Lipinski rule of 5 along with the no. of rotatable bonds and surface area of ligands with Paclitaxel

**ligand**	**Mol. weight**	**LogP**	**#Rotatable bonds**	**# Acceptors**	**#Donors**	**Surface area**
Paclitaxel	853.918	3.7357	10	14	4	357.885
Triterpenoid Compound 1	452.679	6.0384	6	3	2	200.291
Triterpenoid Compound 2	454.625	5.4023	5	3	2	187.561
Triterpenoid Compound 3	520.798	7.6208	9	3	2	231.426

**Table 3:** Comparison of ADMET properties of triterpenoid compounds 1, 2 and 3 with paclitaxel

**Compound name**	**Absorption**	**Disrtibution**	**Metabolism**		**Excretion**	**Toxicity**			
	**Intestinal absorption (human)(% Absorbed)**	**BBB permeability (log BB)**	**CYP2D6 substrate(Yes/No)**	**CYP2D6 inhibitior**	**Total Clearance (log ml/min/kg)**	**AMES toxicity (Yes/No)**	**Oral Rat Acute Toxicity (LD50) mol/kg)**	**Oral Rat Chronic Toxicity (LOAEL)(log mg/kg_bw/day)**	**Hepatotoxicity (Yes/No)**
Paclitaxel	100	-1.731	No	No	0.36	No	2.776	3.393	Yes
Triterpenoid Compound 1	98.782	-0.763	No	No	0.586	No	2.7	2.073	Yes
Triterpenoid Compound 2	98.237	-0.716	No	No	0.552	No	2.579	2.025	Yes
Triterpenoid Compound 3	98.565	-0.942	No	No	0.602	No	3.007	2.309	Yes

**Table 4:** Binding affinity and hydrogen bond interactions of the Paclitaxel and triterpenoids isolated from Cassia fistula with different cancer targets

**Marker type**	**markers**	**Binding affinity**				**Hydrogen bond interactions**			
		**Triterpenoid Compound 1**	**Triterpenoid Compound 2**	**Triterpenoid Compound 3**	**Paclitaxel**	**Triterpenoid Compound 1**	**Triterpenoid Compound 2**	**Triterpenoid Compound 3**	**Paclitaxel**
	BAK	-7.9	-7.9	-8.2	-7.1	-	-	Arg-137, Asp-90, Glu-46, Glu-48	Arg-87, Asn-86, Asp-90, Gln-94
	BAX	-6.1	-6.2	-5.8	-4.4	Asp-157	Ile-155	Glu-156, Leu- 152	Arg-153
	Bcl-2	-8.3	-8.3	-8.7	-7.5	Asp-35, Glu-42	Arg-12, Glu-42	-	Arg-98,Lys-17
Apoptotic	Bcl-xL	-7.8	-8.9	-8.7	-7.9	Tyr-199	-	-	Arg-104, Arg-143
	Caspase-3	-6.8	-7.2	-6.6	-6.8	Gly-145, Gly153	Arg-164	Gly-145, Lys-156, Thr-140	Lys- 137, Lys- 156, Tyr- 37
	Caspase-6	-6.7	-6.9	-6.6	-6.8	Asn-224, Gln-230	Arg- 164	Tyr- 216	Pro-33
	Caspase-8	-8.7	-8.8	-8.3	-9.6	-	Leu-274	-	Arg-1068, Gln-1107, Gln-191
	Caspase-9	-7	-7.3	-7.5	-7.7	-	-	-	Gln-320, Gly-277, Ser- 339
	CDK-4	-8.1	-8.2	-7.7	-8.5	Arg-186	Arg-144	Val- 190	-
	CDK-6	-8.8	-8.9	-8.6	-8.2	-	-	Asp-163	Gly- 239,Phe-283
	Cyclin-D1	-7.2	-7.7	-7.4	-7.8	Cys-73	Arg-140	Arg- 179, Gln- 176, Glu- 75	Thr- 184, Gln-183
Cell cycle	Cylcin-D3	-8.3	-7.8	-7.5	-6.3	Asn-145, Asp- 99,Asp- 158, Lys-35	Arg-38	Thr-277	Phe-287, Ala-286
	P18	-7.4	-7.1	-7	-7.5	-	-	-	-
	P21	-5.3	-5.2	-5	-4.6	Arg-155, Arg 156, Lys-154	Arg-155	Arg- 155	Arg-155
	P27	-7.3	-6.9	-7.7	-7.5	-	Val- 79	-	-
	P52	-6.9	-7	-6.4	-7.0	Ser-161	Lue-228	Arg- 156	Asn-227, Ser-226, Asp-251
NFkB	P65	-7.2	-6.9	-7.3	-7.2	Ile- 224	-	Ser-51	Arg-273, Gln-243, Lys-28
	P100	-7.9	-7.3	-7.1	-9.1	Leu-95, Leu- 117	His- 382	-	Lys-153, Ala-104, Arg-193, Arg-103
	CAT	-9.4	-8.8	-8.4	-7.3	-	-	-	Gln-387, Asn-385, Arg-382
Oxidative stress	SOD	-6.7	-6.8	-7.1	-5.9	-	Arg-69	Arg-143, Glu-133, Gly-141	Gly-141, Arg-143
	Glute	-7.3	-7	-6.9	-7.6	Pro- 165	Gln- 54	Glu-164	Arg-29, Thy-100, Asn-15
WNT complex	LRP	-8.6	-9.7	-10	-8.3	His-962, Glu-701	Val-699, Arg-739	Asp-110	Gln-1182, Arg-1184
	frizzled	-8.2	-8.6	-8.2	-7.3	-	Glu-68	Arg-132, Asp-131, Ala-128	-
